# Supplementary material for: Orthosteric and allosteric modulation of human HCAR2 signaling complex
Source: Nat Commun. 2023 Nov 22;14:7620. doi: 10.1038/s41467-023-43537-z (PMC10665550; doi:10.1038/s41467-023-43537-z)
Supplement: Supplementary file 1 — Supplementary Information [file 41467_2023_43537_MOESM1_ESM.pdf]

Orthosteric and allosteric modulation of human HCAR2 signaling complex

Mao et al.

Supplementary Figures 1-10

Supplementary Table 1

Supplementary Fig.1

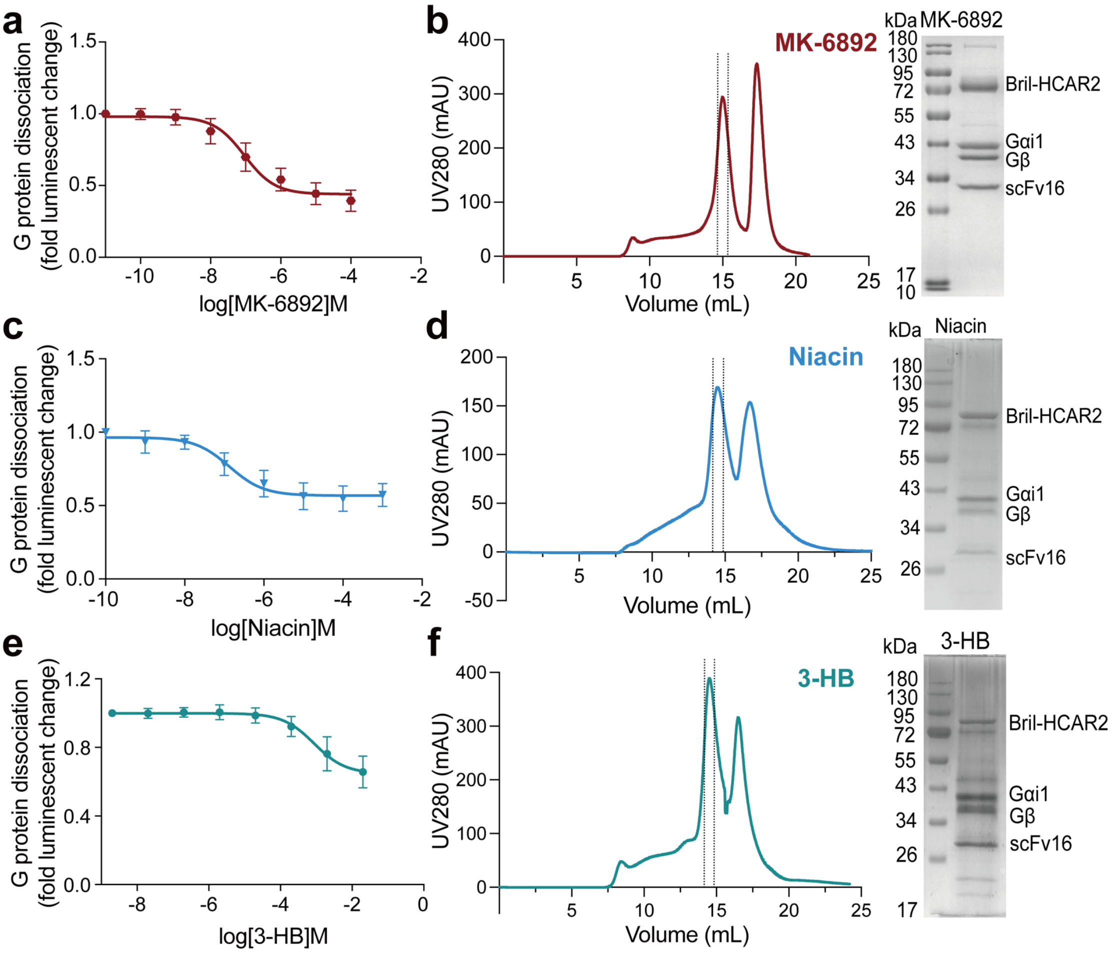

Supplementary Fig.1 | Purification of the HCAR2-Gi1 complexes.

a, c, e Dose-response curves for MK-6892–, Niacin– and 3-HB–induced Gi1 dissociation signal measured by NanoBiT assay, error bars represent the standard deviation of curve fits from a (n = 11), b (n = 9), c (n = 11) independent experiments. Source data are provided as a Source Data file.

b, d, f Size exclusion chromatography profiles and Coomassie blue staining of the purified HCAR2–Gi1 complexes. Samples are prepared and repeated over three times. Source data are provided as a Source Data file.

Supplementary Fig.2

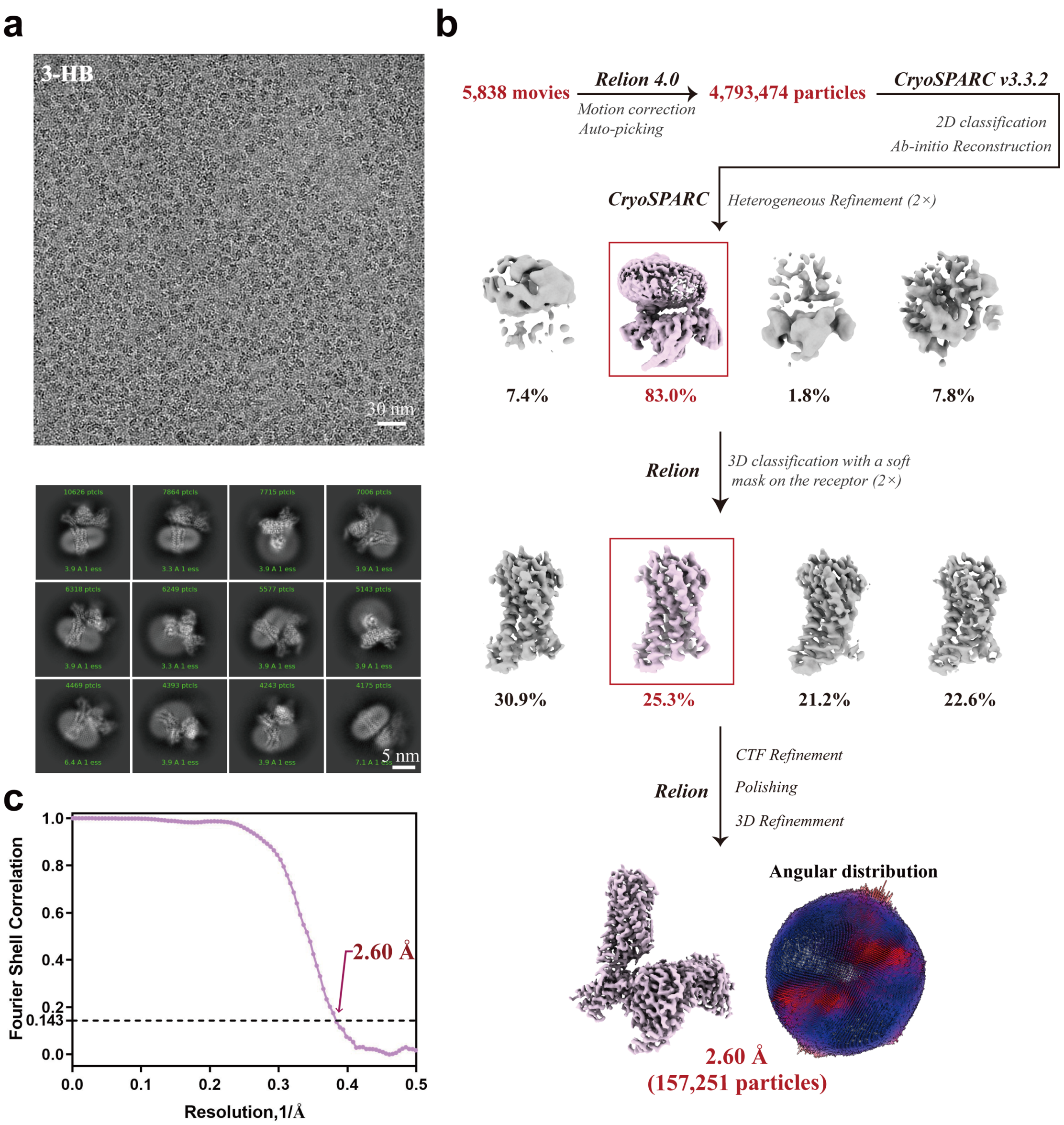

Supplementary Fig.2 | Cryo-EM data processing of the 3-HB/compound 9n bound HCAR2-Gi1 complex.

a Cryo-EM micrographs (scale bar: 30 nm) and 2D class averages (scale bar: 5 nm) of 3-HB/compound 9n–HCAR2–Gi1 complex.

b The flow chart of cryo-EM data processing

c ‘Gold-standard’ Fourier shell correlation curves of the final cryo-EM map.

Supplementary Fig.3

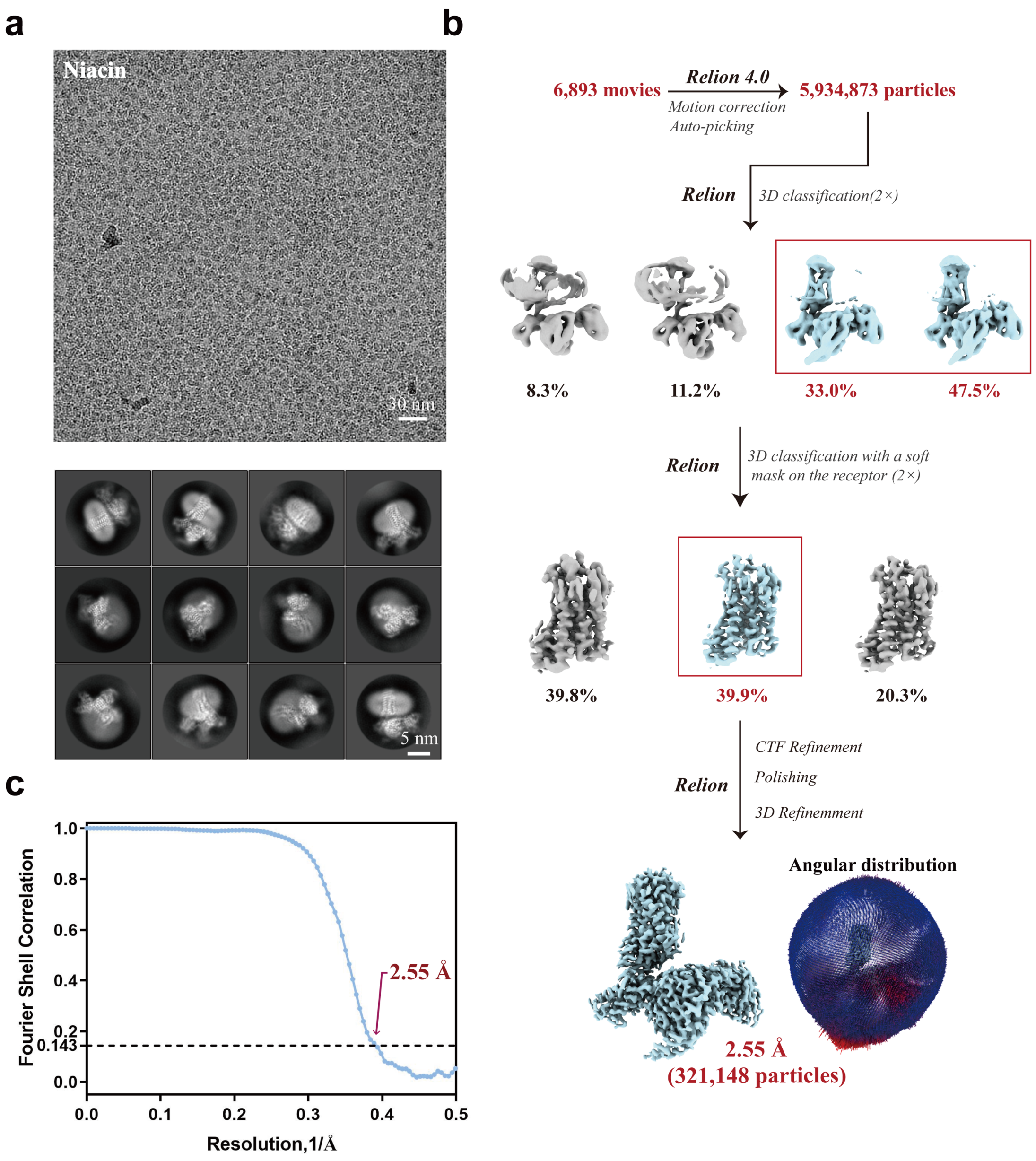

Supplementary Fig.3 | Cryo-EM data processing of the Niacin/compound 9n bound HCAR2-Gi1 complex.

a Cryo-EM micrographs (scale bar: 30 nm) and 2D class averages (scale bar: 5 nm) of Niacin/compound 9n–HCAR2–Gi1 complex.

b The flow chart of cryo-EM data processin.

c 'Gold-standard' Fourier shell correlation curves of the final cryo-EM map.

Supplementary Fig.4

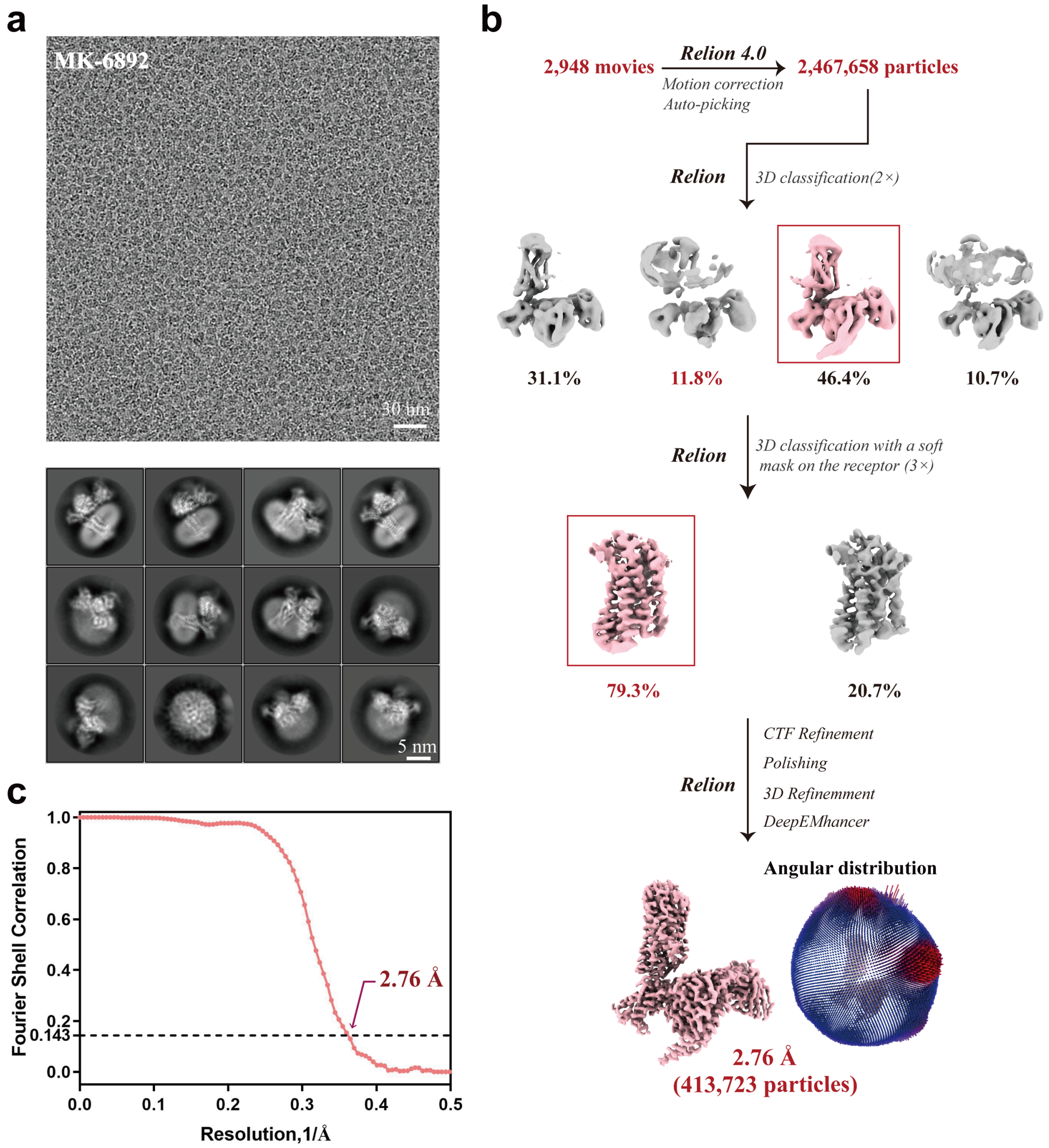

Supplementary Fig.4 | Cryo-EM data processing of the MK-6892 bound HCAR2-Gi1 complex.

a Cryo-EM micrographs (scale bar: 30 nm) and 2D class averages (scale bar: 5 nm) of MK-6892–HCAR2–Gi1 complex.

b The flow chart of cryo-EM data processing.

c ‘Gold-standard’ Fourier shell correlation curves of the final cryo-EM map.

Supplementary Fig.5

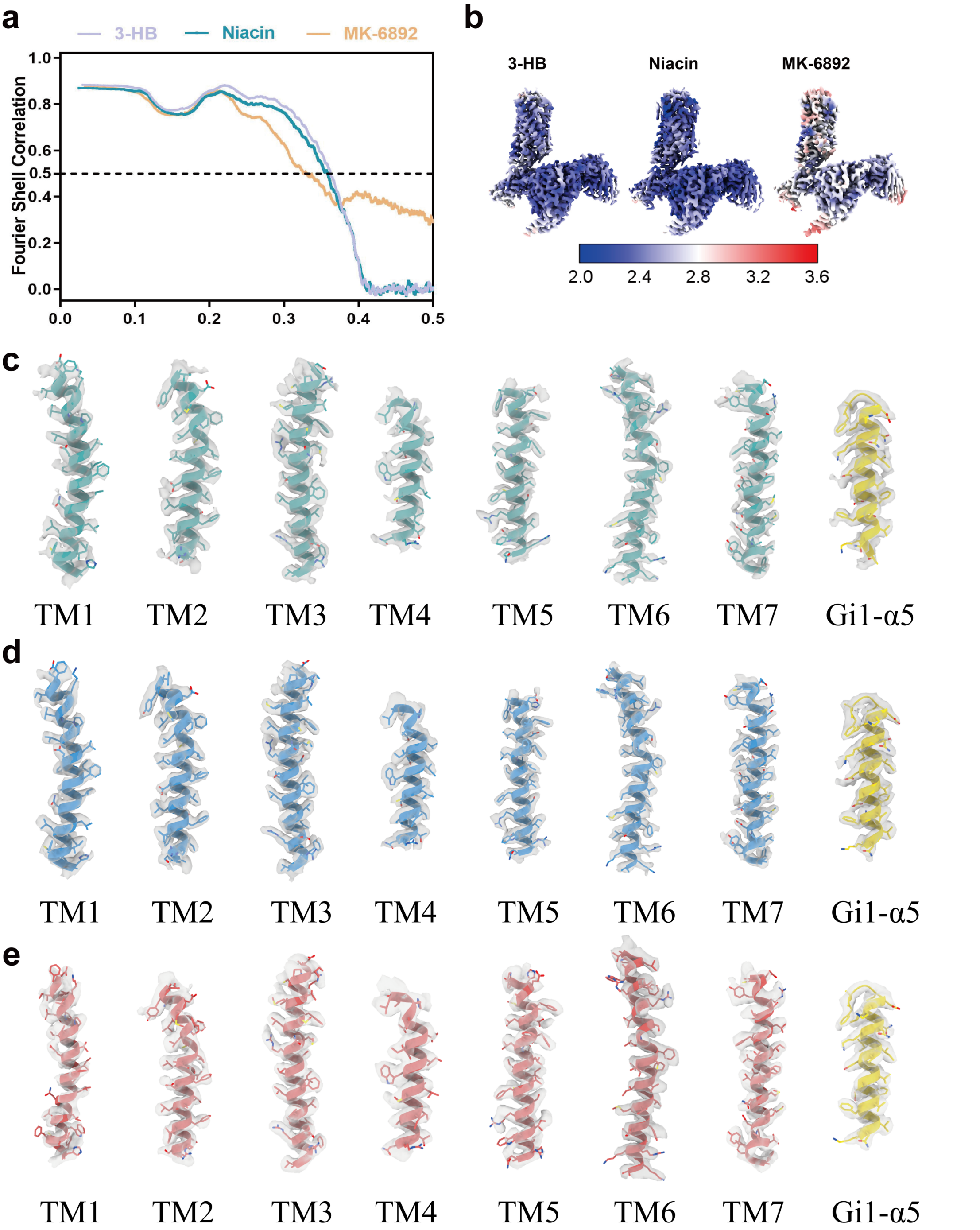

Supplementary Fig.5 | Local resolution and atomic-resolution model of the 3-HB/compound 9n–, Niacin/compound 9n–, and MK-6892–HCAR2–Gi1 complex in the cryo-EM density map.

a Fourier shell correlation curves of the model versus map.

b Cryo-EM maps colored by local resolution.

c, d, e Cryo-EM density maps and models are shown for all seven-transmembrane helices and Gi1 α5-helix of the 3-HB/compound 9n–, Niacin/compound 9n–, and MK-6892–bound HCAR2–Gi1 complexes.

Supplementary Fig.6

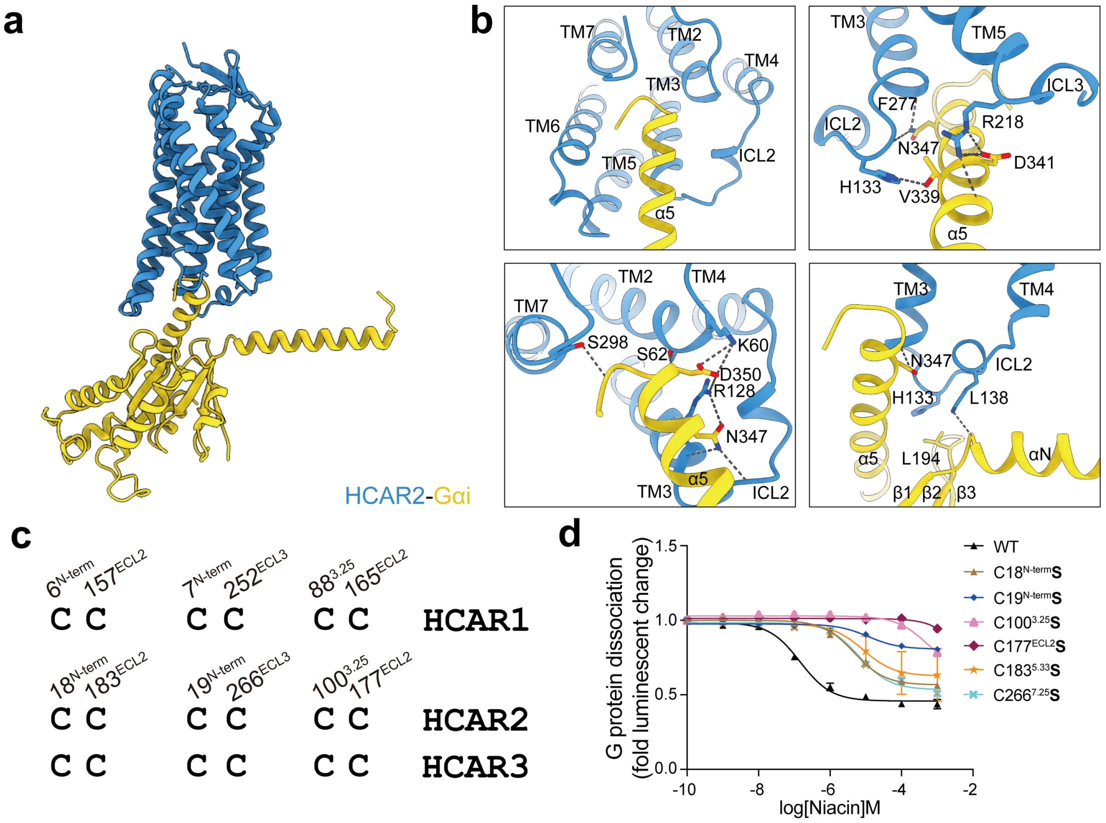

Supplementary Fig.6 | Gi1 coupling and the three conserved disulfide bonds of HCAR2.

a, b Gi1 binding interface of HCAR2. HCAR2 and Gi1 are colored blue and yellow, respectively.

c Sequence alignment of three critical disulfide bonds in HCAR family.

d Dose-response curves of HCAR2 variants harboring mutations in the three disulfide bonds. The Gi1 dissociation signal was detected by NanoBiT assay, error bars represent the standard deviation of curve fits from WT (n = 3), C18$^{N\text{-term}}$S (n = 3), C19$^{N\text{-term}}$S (n = 3), C100$^{N\text{-term}}$S (n = 4), C177$^{N\text{-term}}$S (n = 4), C183$^{N\text{-term}}$S (n = 3) , C266$^{N\text{-term}}$S (n = 3) independent experiments. Source data are provided as a Source Data file.

Supplementary Fig.7

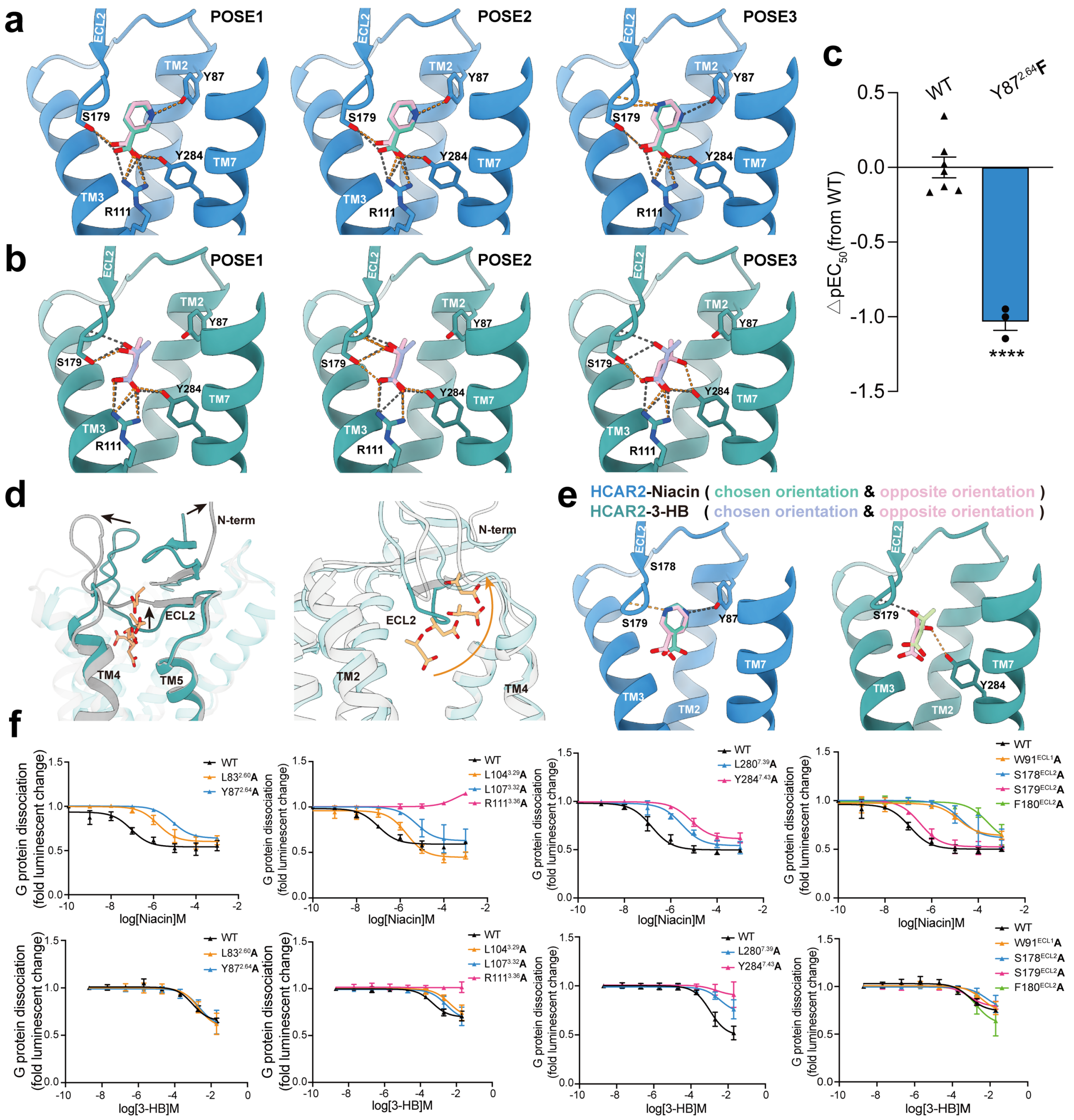

Supplementary Fig.7 | Recognition of 3-HB and Niacin by HCAR2.

a, b Molecular docking analysis of the binding poses of Niacin (a, dark green) and 3-HB (b, purple). The top three docking results (pink) are shown.

c Replacement of Y87$^{2.64}$ with F$^{2.64}$ on Niacin-induced Gi1 dissociation signal. Bars represent differences in calculated agonist potency (pEC50) for each mutant relative to the wild-type receptor (WT). $^{****}P < 0.0001$ (one-way ANOVA followed by Dunnett's multiple comparison test, compared with the response of WT. Data are shown as means ± SEM from three independent experiments). Source data are provided as a Source Data file.

d The potential escaping pathway of 3-HB revealed by MD simulation. As illustrated, the ECL2 exhibits an upward shift from its initial state (deep green) to an intermediate state (gray), thereby creating a crevice by the TM4-TM5-ECL2 region. Within this spatial context, 3-HB (orange) define the plausible pathway for its escape.

e Two potential binding poses of Niacin and 3-HB. Structural analysis demonstrates

that the interacting residues in the two binding modes of the two ligands are almost identical. The primary divergence lies in the hydroxyl group of 3-HB and the pyridinic nitrogen of Niacin, as well as their interactions with the receptor. In our current structure, the hydroxyl group of 3-HB can form hydrogen bonds with both the main chain and side chain of $S179^{ECL2}$ within the receptor. Conversely, in its opposite orientation, only a hydrogen bond can be established with $Y284^{7.43}$. For Niacin, its two distinct poses can each form hydrogen bonds with the main chain of $S179^{ECL2}$ and the side chain of $Y87^{2.64}$. Further functional analysis reveals that the $Y87^{2.64}F$ mutation significantly reduced Niacin's activation.

f Dose-response curves of HCAR2 variants harboring mutations in the Niacin– and 3-HB–binding pocket, error bars represent the standard deviation of curve fits from at least n=3 independent experiments. Source data are provided as a Source Data file.

Supplementary Fig.8

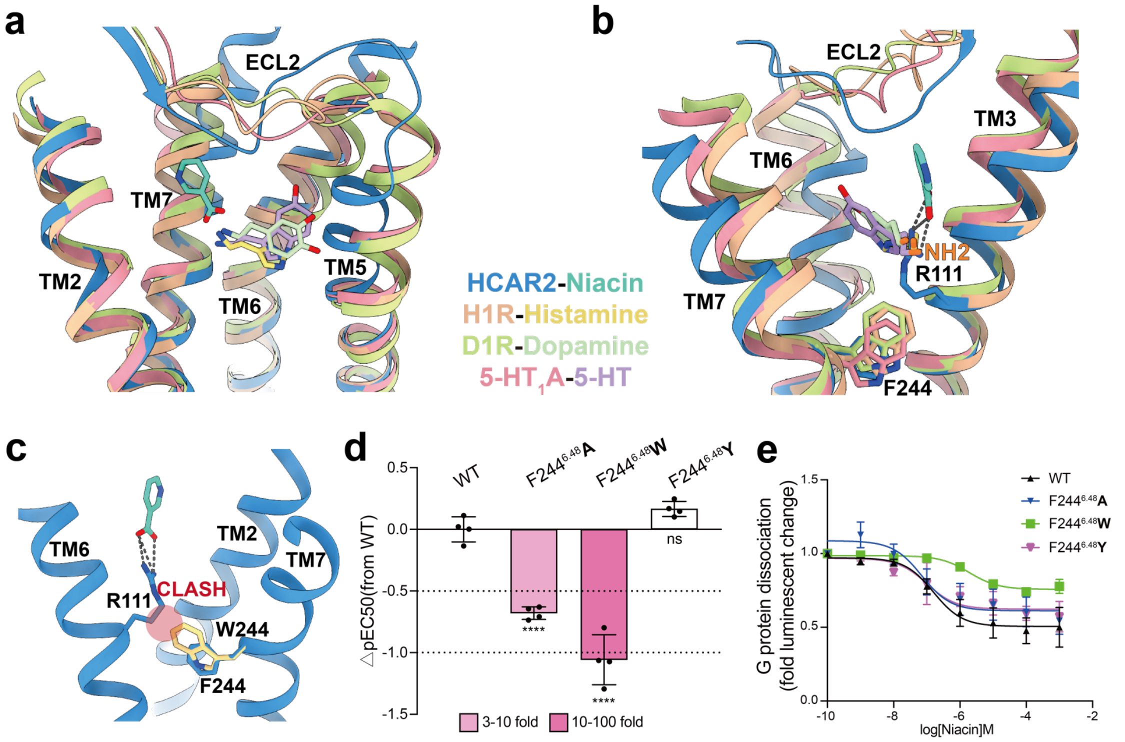

Supplementary Fig.8 | Comparison of ligand binding modes between HCAR2 and aminergic GPCRs.

a, b Superpositions of Niacin/compound 9n (dark green)-bound HCAR2 (blue) with histamine (yellow)- bound H1R (Orange) (PDB: 7DFL), dopamine (light green)-bound DRD1 (green) (PDB: 7LJD) and 5-HT (purple)-bound 5-HT$_1$A (pink) (PDB: 7E2Y). **c** Detailed examination of the non-conserved toggle switch F244$^{6.48}$ in HCAR2. Substitution of F244$^{6.48}$ with W$^{6.48}$ may form steric hindrance with the adjacent R111. **d, e** The effect of F244$^{6.48}$ mutations in HCAR2 on Niacin-induced Gi1 dissociation signal. Data shown are means ± SEM from n=4 independent experiments performed in technical triplicate. $^{ns}P > 0.05$; $^{****}P < 0.0001$ by one-way ANOVA followed by Dunnett's post-test, compared with the response of the WT, the detailed P value for each condition is $P < 0.0001$, $P < 0.0001$, $P = 0.1747$, from left to right. Source data are provided as a Source Data file.

Supplementary Fig.9

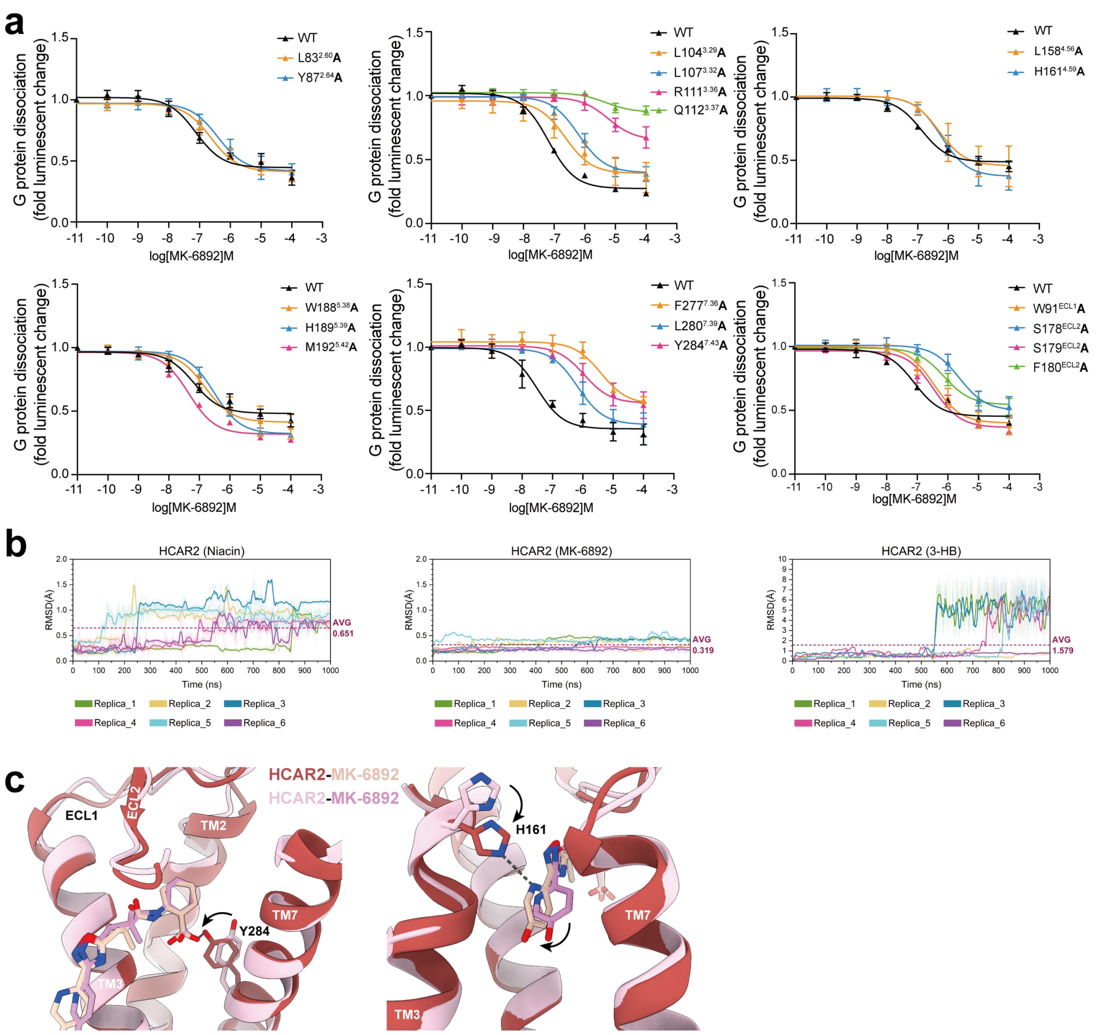

Supplementary Fig.9 | Recognition of MK-6892 by HCAR2.

a Dose-response curves of HCAR2 variants harboring mutations in MK-6892 binding pocket as measured by NanoBiT assay, error bars represent the standard deviation of curve fits from n independent experiments. The number of experiments (n) for each condition is as follows: WT (n = 9), L83$^{2.60}$A (n = 3), Y87$^{2.64}$A (n = 7), W91^{ECL1}A (n = 4), L104$^{3.29}$A (n = 4), L107$^{3.32}$A (n = 5), R111$^{3.36}$A (n = 8), Q112$^{3.37}$A (n = 5), L158$^{4.56}$A (n = 3), H161$^{4.59}$A (n = 6), S178^{ECL2}A (n = 7), S179 ^{ECL2}A (n = 5), F180 ^{ECL2}A (n = 4), W188$^{5.38}$A (n = 7), H189$^{5.39}$A (n = 4), M192$^{5.42}$A (n = 4), F277$^{7.36}$A (n = 4), L280$^{7.39}$A (n = 5), Y284$^{7.43}$A (n = 5). Source data are provided as a Source Data file.

b Molecular dynamics simulations analysis of 3-HB–, Niacin– and MK6892– bound HCAR2, respectively. The structures of 3-HB– and Niacin–bound HCAR2 used in MD analysis contain the compound 9n binding. Simulations were run over 1us and 6 simulations per condition.

c Structural comparison of our resolved MK-6892-bound HCAR2 (red) with the recently reported structure (PDB 7XK2; pink). Our higher-resolution structure offers a more precise depiction of the ligand binding interactions. First, our structure shows a 2.9Å movement of the side-chain of Y284$^{7.43}$ towards the agonist, establishing a hydrogen bond with the carboxyl group of MK-6892. Second, compared to recent

structure, our structure demonstrates a rotation of approximately 90 degrees in the hydroxypyridine moiety of MK-6892, resulting in hydrophobic and hydrogen-bonding interactions with $H161^{4.59}$.

Supplementary Fig.10

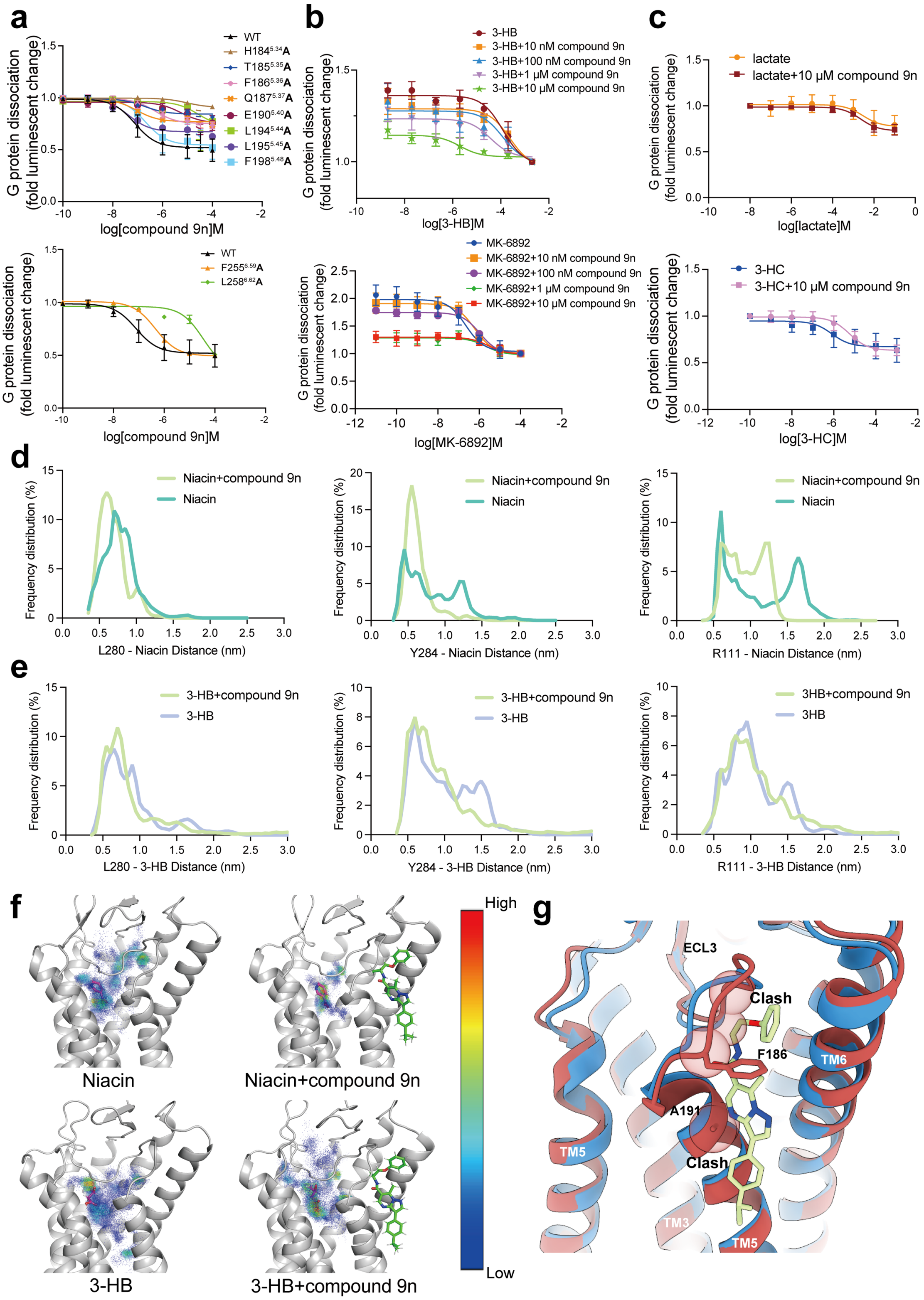

Supplementary Fig.10 | Recognition of compound 9n by HCAR2.

a Dose-response curves of HCAR2 variants harboring mutations in the compound 9n-binding pocket, error bars represent the standard deviation of curve fits from n independent experiments. The number of experiments (n) for each condition is as follows: WT (n = 8), $H184^{5.34}A$ (n = 3), $T185^{5.35}A$ (n = 3), $F186^{5.36}A$ (n = 4), $Q187^{5.37}A$ (n = 3), $E190^{5.40}A$ (n = 6), $L194^{5.44}A$ (n = 3), $L195^{5.45}A$ (n =3), $F198^{5.48}A$ (n = 6),

F255$^{6.59}$A (n = 4), L258$^{6.62}$A (n = 3).

b Dose-response curves for the compound 9n-potentiated 3-HB (top) and MK-6892 (bottom) activation, error bars represent the standard deviation of curve fits from at n independent experiments. The number of experiments (n) for each condition is as follows: 3-HB (n = 3), 3-HB+10 nM compound 9n (n = 4), 3-HB+100 nM compound 9n (n = 6), 3-HB+1 μM compound 9n (n = 6), 3-HB+10 μM compound 9n (n = 6), MK-6892 (n = 3), MK-6892+10 nM compound 9n (n = 3), MK-6892+100 nM compound 9n (n = 3), MK-6892+1 μM compound 9n (n = 3), MK-6892+10 μM compound 9n (n = 3).

c Dose-response curves of lactate (top) and 3-HC (bottom) induced Gi1 signaling in the absence or presence of compound 9n, error bars represent the standard deviation of curve fits from n independent experiments. The number of experiments (n) for each condition is as follows: lactate (n = 4), lactate+10 μM compound 9n (n = 6), 3-HC (n = 6) , lactate+10 μM compound 9n (n = 3). Source data are provided as a Source Data file.

d Frequency distribution of distances between Niacin and L280$^{7.39}$, Y284$^{7.43}$, R111$^{3.36}$ in the presence (light green) and absence (cyan) of compound 9n, respectively.

e Frequency distribution of distances between 3-HB and L280$^{7.39}$, Y284$^{7.43}$, R111$^{3.36}$ in the presence (light green) and absence (lavender) of compound 9n, respectively.

f Frequency distribution plot of agonists centroids with or without compound 9n (light green). Frequency distribution is colored by spectrum from high (red) to low (blue), initial positions of agonists are shown in magenta.

g Structural superimposition of the Niacin and compound 9n (light green) -bound HCAR2 (blue) with the MK-6892-bound complex (red) showed that MK-6892-induced outward movement of TM5 would create steric hindrance with compound 9n. Source data are provided as a Source Data file.

Supplementary Table.1 | Cryo-EM data collection, model refinement and validation statistics.

	3-HB & Compound 9n	Niacin & Compound 9n	MK-6892
**Data collection and processing**			
Magnification	150,540	150,540	4,9310
Voltage (kV)	300	300	300
Electron exposure ($e^{-}/Å^2$)	52	52	64
Defocus range (μm)	-1.0 ~ -2.5	-1.0 ~ -2.5	-1.0 ~ -2.5
Pixel size (Å)	0.93	0.93	1.014
Symmetry imposed	C1	C1	C1
Initial particle projections (no.)	4,793,474	5,934,873	2,467,658
Final particle projections (no.)	157,251	321,148	413,723
Map resolution (Å)	2.60	2.55	2.76
FSC threshold	0.143	0.143	0.143
Map resolution range (Å)	2.2-4.0	2.2-4.0	2.4-4.0
**Refinement**			
Initial model used	AF2 & 7WIC	AF2 & 7WIC	AF2 & 7WIC
Model resolution (Å)	2.7	2.8	3.0
FSC threshold	0.5	0.5	0.5
Model resolution range (Å)	2.4-4.0	2.4-4.0	2.6-4.0
Map sharpening *B* factor ($Å^2$)	-85.51	-89.28	DeepEMhancer
Model composition			
Non-hydrogen atoms	9,187	9,189	9120
Protein residues	1,151	1,151	1151
B factors ($Å^2$)			
Protein	38.58	41.49	58.15
Ligand	53.47	49.90	63.28
R.m.s. deviations			
Bond lengths (Å)	0.003	0.004	0.003
Bond angles (°)	0.669	0.747	0.609
Validation			
MolProbity score	1.53	1.61	1.46
Clashscore	7.06	8.21	6.08
Rotamer outliers (%)	0.00	0.00	0.00
Ramachandran plot			
Favored (%)	97.27	97.10	97.36
Allowed (%)	2.73	2.73	2.64
Disallowed (%)	0.00	0.18	0.00
