# Supplementary material for: Orthosteric and allosteric modulation of human HCAR2 signaling complex
Source: Nat Commun. 2023 Nov 22;14:7620. doi: 10.1038/s41467-023-43537-z (PMC10665550; doi:10.1038/s41467-023-43537-z)
Supplement: Supplementary file 3 — Description of Additional Supplementary Files [file 41467_2023_43537_MOESM3_ESM.pdf]

Description of Additional Supplementary Files

Supplementary Data 1: The Gi1 dissociation assays of the HCAR2 variants harboring mutations of the residues involved in ligand recognition and activation.
[a]Data shown are means ± SEM from at least three independent experiments performed in technical triplicate. nd, not determined; $^{ns}P > 0.05$; $^{*}P < 0.05$; $^{**}P < 0.01$ $^{***}P < 0.001$ and $^{****}P < 0.0001$ by one-way ANOVA followed by Dunnett's post-test, compared with the response of the WT. [b] The span is defined as the window between the maximal response (Emax) and the vehicle.

Supplementary Data 2: Cell surface expression of wild-type and mutant HCAR2, related to Figures 2, 3, 4.
[a]Data shown are means ± SEM from at least three independent experiments performed in technical triplicate.

Supplementary Data 3: List of primer sequences used in this study.

Supplementary Data 4: System setup for MD simulations.

Supplementary Data 5: The mdp file contain all configuration used for MD production simulations. All initial and final coordinates of MD simulations were supplied as gro files.
